# Clinical Performance of Radiofrequency Ablation for Treatment of Uterine Fibroids: Systematic Review and Meta-Analysis of Prospective Studies

**DOI:** 10.1089/lap.2019.0550

**Published:** 2019-11-08

**Authors:** Linda D. Bradley, Resad P. Pasic, Larry E. Miller

**Affiliations:** ^1^ Department of Obstetrics and Gynecology, Women's Health Institute, Center for Menstrual Disoders, Cleveland Clinic, Cleveland, Ohio.; ^2^ Department of Obstetrics and Gynecology, University of Louisville Hospital, Louisville, Kentucky.; ^3^ Miller Scientific Consulting, Inc., Asheville, North Carolina.

**Keywords:** laparoscopic, leiomyoma, myoma, radiofrequency, transcervical, transvaginal

## Abstract

***Background:*** Radiofrequency ablation (RFA) has emerged as a safe and effective treatment option for women with symptomatic uterine fibroids and can be delivered by laparoscopic, transvaginal, or transcervical approaches. The evidence regarding typical patient outcomes with RFA has not previously been examined in a comprehensive fashion.

***Materials and Methods:*** We performed a systematic review of prospective studies for treatment of uterine fibroids with RFA. Main outcomes were procedure time, patient recovery metrics, change in fibroid volume, symptom severity score (SSS), health-related quality of life (HRQL), and reinterventions. Data were analyzed with random effects meta-analysis and metaregression.

***Results:*** We identified 32 articles of 1283 unique patients (median age: 42 years) treated with laparoscopic RFA (19 articles), transvaginal RFA (8 articles), or transcervical fibroid ablation (5 articles). Mean procedure time was 49 minutes, time to discharge was 8.2 hours, time to normal activities was 5.2 days, and time to return to work was 5.1 days. At 12 months follow-up, fibroid volume decreased by 66%, HRQL increased by 39 points, and SSS decreased by 42 points (all *P* < .001 versus baseline). The annual cumulative rate of reinterventions due to fibroid-related symptoms was 4.2%, 8.2%, and 11.5% through 3 years.

***Conclusions:*** RFA of uterine fibroids significantly reduces fibroid volume, provides significant durable improvements in fibroid-related quality of life, and is associated with favorable reintervention rates.

## Introduction

Uterine fibroids are the most common benign solid pelvic tumor in women, developing in ∼70% to 80% of women by 50 years of age.^1^ More than 1 in 3 women with uterine fibroids report symptoms that interfere with activities of daily living such as heavy menstrual bleeding and/or bulk symptoms.^2^ Self-management with nonprescription medication or lifestyle modification is common, but often unsuccessful.^3^ Several surgical and interventional treatments are available to women with persistent symptoms attributable to uterine fibroids, including hysterectomy, myomectomy, and uterine artery embolization. However, patient acceptance of these treatments may be limited due to the increasing demand for less invasive therapies that preserve the uterus.^3^

Radiofrequency ablation (RFA) has emerged as a safe and effective treatment alternative as the procedure can be delivered in a minimally invasive fashion. RFA may be delivered by a laparoscopic, transvaginal, or transcervical approach into the uterine fibroid to induce coagulative necrosis^4^ with subsequent reduction in fibroid-related symptoms. Previous reviews, often limited to a single device or treatment route, have reported patient outcomes following laparoscopic RFA.^5,6^ To the authors' knowledge, no systematic review has evaluated the clinical utility of each RFA delivery approach for the treatment of uterine fibroids. We hypothesized that RFA would provide significant decreases in fibroid volume and improvements in quality of life for women with symptomatic uterine fibroids. The primary aim of this study was to report the effectiveness of RFA for symptomatic uterine fibroids by means of a systematic review and meta-analysis.

## Materials and Methods

### Eligibility criteria and search strategy

The conduct, analysis, and reporting of this systematic review adhered to the Preferred Reporting Items for Systematic Reviews and Meta-analyses (PRISMA).^7^ Prospective studies of RFA for symptomatic uterine fibroid treatment were eligible for inclusion in this systematic review. We considered randomized trials, comparative cohort studies, and noncomparative cohort studies for this review, and extracted data only from the RFA arms of the study. We excluded case reports and studies with less than 10 patients, studies in which patients received concomitant surgeries due to a potential for confounding of patient outcomes, and studies that reported no main outcomes. No date or language restrictions were applied to the searches. We performed systematic searches of Medline, Embase, and the Cochrane Central Register of Controlled Trials for potentially eligible studies. Additional searches were conducted in the Directory of Open Access Journals and Google Scholar. Manual searches of the reference lists of included articles and relevant meta-analyses were performed. The search strategy included combinations of anatomic-, diagnosis-, and treatment-specific keywords. The Medline search strategy is provided in Supplementary Table S1; the search strategy for other databases was adapted as necessary.

Two researchers with expertise in systematic reviews independently screened titles and abstracts for eligibility. Full-text articles were obtained for all potentially relevant studies. To account for multiple articles derived from the same primary study or subsamples of the primary study, we preferentially extracted data from the article reporting the longest follow-up duration on the entire cohort and supplemented any missing data using other articles derived from that study. Thus, all reported data were derived from unique patients. Disagreements related to study eligibility were resolved by discussion and consensus. The final searches were performed on May 31, 2019.

### Data extraction

Researchers independently extracted data from eligible studies using standardized data collection forms. For each study, we recorded metadata, patient characteristics, study characteristics, treatment regimens, and main outcomes. Main outcomes included procedure time, length of stay, time to normal activities, time to return to work, change in uterine fibroid volume, change in symptom severity score (SSS) and health-related quality of life (HRQL) on the Uterine Fibroid Symptom Health-Related Quality of Life Questionnaire (UFS-QoL),^8^ and surgical reinterventions.

We extracted fibroid volume, SSS, and HRQL data at baseline, 3 months, 6 months, 12 months, and beyond 12 months, where the last interval consisted of the latest follow-up interval beyond 12 months reported in each study. The rate of surgical reinterventions for fibroid-related symptoms was calculated at 6 months and annually thereafter through 3 years. Reinterventions were conservatively assumed to be performed for fibroid-related symptoms unless explicitly stated otherwise in the article. We used the National Institute of Health assessment tool for before/after studies to evaluate the methodological quality of eligible studies.^9^ Data extraction discrepancies between the two researchers were resolved by discussion and consensus.

### Statistical analyses

Procedure time, length of stay, time to normal activities, and time to return to work were reported using the weighted mean statistic. Change in uterine fibroid volume was reported as a weighted percent change from baseline. Change in SSS and HRQL was reported using the weighted mean difference relative to baseline. The surgical reintervention rate was reported as a weighted event rate. Outcome estimates were calculated for each study and the overall pooled result was reported along with the 95% confidence interval (CI). We prospectively specified an inverse variance random effects model for all analyses given the variation among study designs and methods of RFA delivery. We evaluated temporal trends in fibroid volume, UFS-QoL scores, and reinterventions by pooling data at distinct follow-up intervals.

We estimated heterogeneity among studies with the *I*^2^ statistic, where a value of 0% represented no heterogeneity and larger values represented increasing heterogeneity.^10^ In accordance with Cochrane Collaboration recommendations, we performed metaregression analysis for any outcome reported in at least 10 studies. We tested the robustness of the meta-analysis conclusions with three sensitivity analysis, including a reanalysis using a fixed-effects meta-analysis model, a one-study removed analysis where the meta-analysis was recalculated following iterative one-at-a-time removal of each study, and reanalysis of only the studies with the highest methodological quality. *P*-values were two sided with a significance level <.05. Analyses were performed using Stata v14.2 (StataCorp).

## Results

### Study selection

**FIG. 1.**
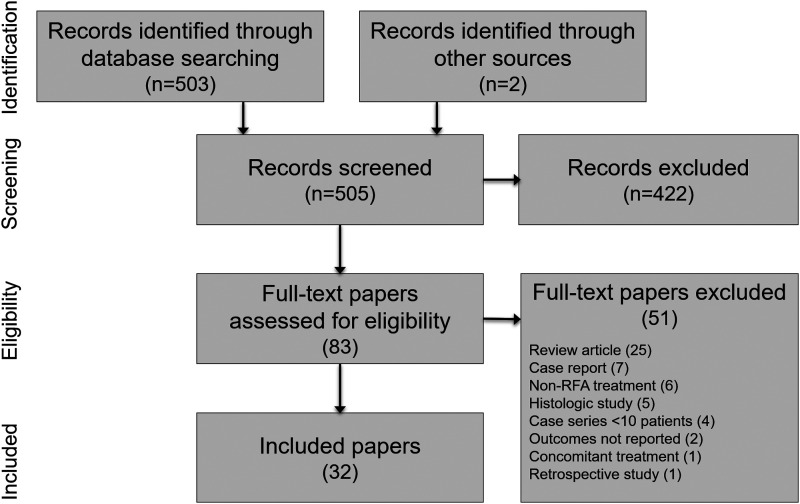
PRISMA study flow diagram. PRISMA, Preferred Reporting Items for Systematic Reviews and Meta-analyses; RFA, radiofrequency ablation.

We identified 505 articles in our searches and ultimately included 32 articles of 1283 unique patients treated with RFA for uterine fibroids in this systematic review. A PRISMA flow diagram depicting the study identification and selection process is provided in Figure 1. Among the full-text articles that were reviewed, 51 were excluded, with review articles (25), case reports (7), and non-RFA treatments (6) the most common reasons (Supplementary Table S2).

### Patient and study characteristics

Baseline patient characteristics in each study are reported in Table 1. Among included studies, mean patient age ranged from 39 to 45 years (median 42 years), the number of treated fibroids ranged from 1 to 5 (median 1.7) per patient, and fibroid volume ranged from 10 to 305 cm^3^ (median 74 cm^3^). Baseline UFS-QoL scores ranged from 22 to 77 for HRQL (median 49) and 32 to 76 for SSS (median 55). Study design characteristics are reported in Table 2. Among the 32 articles, 19 reported laparoscopic RFA, 8 reported transvaginal RFA, and 5 reported transcervical fibroid ablation (TFA). RFA was delivered using ultrasound guidance in 90% of the studies. Patient follow-up in each study ranged from in-hospital to 5.3 years (median 12 months).

**Table 1.** Patient Characteristics in Studies Included in the Meta-Analysis

*Study*	*Age (years)*	*Patients desiring future fertility*	*Fibroid types (%)*					*No. of treated fibroids per patient*	*Total fibroid volume (cm^3^)*	*HRQL*^a^	*SSS*^a^
			*Submucous*	*Intramural*	*Subserous*	*Transmural*	*Unspecified*				
Bongers et al.^15^	43^b^	Excluded	61	30	0	9	0	1.8 ± 1.1	10 (<1, 77)^c^	34 ± 19	62 ± 17
Brölmann et al.^16^											
Garza-Leal^17^	43^b^	Excluded	49	46	0	6	0	2.1	—^d^	27 ± 22	65 ± 17
Braun et al.^18^	40 ± 7	Included	4	35	49	5	7	4.2 ± 3.3	—^d^	—	—
Brucker et al.^19^	40 ± 8	Included	0	49	51	0	0	2.9 ± 2.6	—^d^	77	40
Hahn et al.^20^											
Krämer et al.^21^											
Carrafiello et al.^22^	40 (27, 51)^e^	Excluded	9	45	0	0	45	1 (1, 1)^c^	102 (45, 278)^e^	62 (37, 86)^e^	50 (32, 67)^e^
Cho et al.^23^	43 ± 4	Excluded	0	0	0	0	100	—	65 ± 13	59 ± 16	49 ± 11
Cho et al.^24^	40 ± 7	Included	100	0	0	0	0	—	112 ± 53	46 ± 13	76 ± 9
Chudnoff et al.^25^	43 ± 5	Excluded	21	46	25	5	2	5.0 ± 4.4	80 ± 84	39 ± 19	60 ± 19
Galen et al.^26^											
Guido et al.^27^											
Berman et al.^28^											
Chudnoff et al.^29^	43 (31, 50)^c^	Excluded	21	49	10	21	0	3.0 ± 2.1	71 ± 85	40 ± 21	55 ± 19
Miller and Osman^30^											
Galen et al.^31^	42 ± 6	Excluded	6	50	38	3	3	3 (1, 20)^c^	—	49 ± 24	54 ± 23
Garza-Leal et al.^32^											
Robles et al.^33^											
Ghezzi et al.^34^	42 (40, 50)^c^	Excluded	0	100	0	0	0	1 (1, 3)^c^	77 (15, 333)^c^	63 (23, 94)^c^	44 (13, 91)^c^
Bergamini et al.^35^											
Iversen and Dueholm^36^	45 ± 7	Excluded	20	80	0	0	0	1 (1, 3)^c^	123 (24, 675)^c^	22 ± 21	61 ± 17
Iversen et al.^37^											
Jiang et al.^38^	41 ± 6	Included	9	78	13	0	0	1 (1, 3)^c^	60 (7, 321)^c^	72 ± 13	32 ± 15
Kim et al.^39^	40 ± 7	Included	6	0	0	0	94	2 (1, 3)^c^	305 (48, 1022)^e^	—	57 ± 21
Lee et al.^40^	45 (42, 51)^e^	Excluded	100	0	0	0	0	1 (1, 1)^c^	58^b^	49 ± 15	71 ± 8
Marcos et al.^41^	44 ± 5	Included	0	71	29	0	0	1 (1, —)^c^	112 ± 65	—	—
Meng et al.^42^	39 ± 6	Excluded	13	66	21	0	0	1 (1, 3)^c^	70 ± 59	—	—
Rattray et al.^43^	39 ± 7	Included	0	0	0	0	100	3.4 ± 2.4	—^d^	40 ± 26	62 ± 20
Rey et al.^44^	39 ± 9	Included	100		0	0	0	—	122 ± 183	—	—
Turtulici et al.^45^	45 ± 8	Excluded	0	0	0	0	100	1.7 (1, 3)^e^	14 (5, 42)^e^	68 ± 36	—
Wu et al.^46^	41 (32, 52)^c^	Excluded	21	79	0	0	0	1.2 (1, 3)^e^	32 (1, 78)^c^	65 ± 41	45 ± 34

^a^ Derived from the UFS-QoL questionnaire.

^b^ Estimated.

^c^ Median (min, max).

^d^ Diameter reported only.

^e^ Mean (min, max).

HRQL, health-related quality of life; SSS, symptom severity score; UFS-QoL, Uterine Fibroid Symptom Health-Related Quality of Life Questionnaire.

**Table 2.** Design Characteristics of Studies Included in the Meta-Analysis

*Study*	*Study ID*	*Treatment period*	*Number and location of sites*	*No. of patients*	*RFA trade name (manufacturer)*	*RFA delivery*	*Follow-up (months)*
Bongers et al.^15^	FAST-EU; NCT01226290	—, 2013^a^	7 Intercontinental	50	Sonata (Gynesonics)	TFA; US guidance	12 (6, 12)^b^
Brölmann et al.^16^							
Garza-Leal^17^	VITALITY	—	1 Mexico	17	Sonata (Gynesonics)	TFA; US guidance	64 (57, 73)^b^
Braun et al.^18^	TRUST postmarket	2014, 2016	4 United States, Canada	40	Acessa (Acessa Health)	PL; US guidance	2 (0, 2)^b^
Brucker et al.^19^	NCT01750008	2012, 2013	1 Germany	25	Acessa (Acessa Health)	PL; US guidance	21 (—, 24)^b^
Hahn et al.^20^							
Krämer et al.^21^							
Carrafiello et al.^22^	—	2006, 2008	1 Italy	11	RF3000 (Boston Scientific)	PL; US guidance	9 (3, 12)^c^
Cho et al.^23^	—	2004, 2006	1 Korea	153	M-1004 (RF Medical System)	TV; US guidance	— (—, 18)^b^
Cho et al.^24^	—	2009, 2012	1 Korea	24	M-1004 (RF Medical System)	TV	— (—, 24)^b^
Chudnoff et al.^25^	Halt Phase III; NCT00874029	2009, 2011	9 United States, Latin America	135	Acessa (Acessa Health)	PL; US guidance	31 (—, 36)^b^
Galen et al.^26^							
Guido et al.^27^							
Berman et al.^28^							
Chudnoff et al.^29^	SONATA; NCT02228174	2015, 2016	22 United States, Mexico	147	Sonata (Gynesonics)	TFA; US guidance	22 (—, 24)^b^
Miller and Osman^30^							
Galen et al.^31^	Halt Phase II	—, 2008^a^	2 Latin America	69	Acessa (Acessa Health)	PL; US guidance	11 (3, 12)^b^
Garza-Leal et al.^32^							
Robles et al.^33^							
Ghezzi et al.^34^	—	2003, 2005	3 Italy	25	Model 1500X (AngioDynamics)	PL	24 (12, 36)^c^
Bergamini et al.^35^							
Iversen and Dueholm^36^	—	2007, 2010	2 Norway, Denmark	66	Model 1500X (AngioDynamics)	PL; US guidance	59 (37, 74)^b^
Iversen et al.^37^							
Jiang et al.^38^	—	2009, 2012	— China	46	BBT-RF-B (Ban Bian Tian)	TV; US guidance	— (—, 18)^b^
Kim et al.^39^	—	2004, 2008	— Korea	69	M-2004 (RF Medical System)	TV; US guidance	— (—, 12)^b^
Lee et al.^40^	—	2005, 2007	1 Korea	58	M-1004 (RF Medical System)	TV; US guidance	— (—, 18)^b^
Marcos et al.^41^	—	2011, 2012	1 Spain	17	RF3000 (Boston Scientific)	PL; US guidance	6 (—, 6)^b^
Meng et al.^42^	—	2009, 2009	1 China	50	Cool-Tip (Medtronic)	PL; US guidance	<1^d^
Rattray et al.^43^	TRUST; NCT015663783	2012, 2017	≥2 Canada	23	Acessa (Acessa Health)	PL; US guidance	— (—, 3)^b^
Rey et al.^44^	—	2015, 2017	1 Spain	205	VIVA (STARmed)	TV; US guidance	— (—, 12)^b^
Turtulici et al.^45^	—	2017, 2018	1 Italy	19	VIVA (STARmed)	TV; US guidance	6 (6, 6)^b^
Wu et al.^46^	—	2010, 2012	1 China	51	DS98F-D (Huanghe)	TV; US guidance	11 (6, 12)^b^

^a^ Estimated.

^b^ Mean (min, max).

^c^ Median (min, max).

^d^ In-hospital follow-up only.

PL, percutaneous laparoscopic; RFA, radiofrequency ablation; TFA, transcervical fibroid ablation; TV, transvaginal; US, ultrasound.

Among the 20 prospective primary studies in this review (reported in 32 articles), study quality was rated as good or fair for 19 of 20 studies. The study design elements that were most frequently missing from published reports were interrupted time-series design (20 of 20 studies), blinded outcome assessors (20 of 20 studies), analyses that failed to adjust for attrition (19 of 20 studies), and no justification for sample size (15 of 20 studies) (Supplementary Table S3).

### Procedure and recovery results

The weighted mean procedure time was 49 minutes (95% CI: 41–56 minutes). Procedure time was significantly different among RFA delivery approaches (laparoscopic, 73 minutes; TFA, 44 minutes; transvaginal, 24 minutes), where all pairwise comparisons were *P* ≤ .002. Time to discharge, time to normal activities, and time to return to work were reported inconsistently and, therefore, comparisons of RFA delivery approaches were reported descriptively only. The weighted mean time to discharge was 8.2 hours (95% CI: 6.3–10.0 hours), including 10.7 hours for laparoscopic RFA, 2.5 hours for TFA, and 2.5 hours for transvaginal RFA. The weighted mean time to normal activities was 5.2 days (95% CI: 3.3–7.1 days), including 9.0 days for laparoscopic RFA, 3.3 days for TFA, and no studies for transvaginal RFA. The weighted mean return to work was 5.1 days (95% CI: 3.7–6.5 days), including 6.5 days for laparoscopic RFA, 3.6 days for TFA, and no studies for transvaginal RFA (Table 3). Substantial heterogeneity among studies was observed for each of these outcomes, with *I*^2^ ranging from 85% to >99% (all *P* < .001).

**Table 3.** Procedure and Recovery Results of Studies Included in the Meta-Analysis

*Study*	*Procedure time (minutes)*	*Time to discharge (hours)*	*Time to return to normal activities (days)*	*Time to return to work (days)*
Bongers et al.^15^	39 ± 23	—	4.4 ± 3.1	—
Brölmann et al.^16^				
Garza-Leal^17^	—	—	—	—
Braun et al.^18^	114 ± 60	6.8 ± 3.2	—	—
Brucker et al.^19^	66 ± 24	10.0 ± 5.5	20.5 (5, 103)^a^	10.0 (2, 86)^a^
Hahn et al.^20^				
Krämer et al.^21^				
Carrafiello et al.^22^	20 (15, 25)^b^	—	—	—
Cho et al.^23^	— (10, 40)	—	—	—
Cho et al.^24^	—	—	—	—
Chudnoff et al.^25^	126 ± 60	—	9.0 (2, 60)^a^	5.0 (0, 29)^a^
Galen et al.^26^				
Guido et al.^27^				
Berman et al.^28^				
Chudnoff et al.^29^	47 ± 30	2.5 ± 1.2	2.2 ± 2.2	3.6 ± 2.6
Miller and Osman^30^				
Galen et al.^31^	140 (42, 290)^a^	—	4.5 (1, 11)^b^	4.0 (2, 10)^a^
Garza-Leal et al.^32^				
Robles et al.^33^				
Ghezzi et al.^34^	25 (20, 45)^a^	18^c^	—	—
Bergamini et al.^35^				
Iversen and Dueholm^36^	—	—	—	—
Iversen et al.^37^				
Jiang et al.^38^	25 (20, 30)^b^	—	—	—
Kim et al.^39^	18 ± 5	—	—	—
Lee et al.^40^	—	—	—	—
Marcos et al.^41^	36 ± 11	12.0 (8, 24)^b^	—	—
Meng et al.^42^	—	—	—	—
Rattray et al.^43^	73 ± 26	6.7 ± 3.0	—	11.1 ± 7.6
Rey et al.^44^	17 (11, 44)^b^	—	—	—
Turtulici et al.^45^	28 (16, 43)^b^	—	—	—
Wu et al.^46^	— (20, 40)	2.5^c^	—	—
**POOLED RESULT^d^**	49 (41–56)	8.2 (6.3–10.0)	5.2 (3.3–7.1)	5.1 (3.7–6.5)
***Laparoscopic RFA***	73 (56–91)	10.7 (5.9–15.5)	9.0 (3.8–14.1)	6.5 (3.8–9.2)
***Transvaginal RFA***	24 (20–28)	2.5 (2.4–2.6)	—	—
***TFA***	44 (36–51)	2.5 (2.3–2.7)	3.3 (1.1–5.4)	3.6 (3.1–4.1)

^a^ Median (min, max).

^b^ Mean (min, max).

^c^ Estimated value.

^d^ Pooled results derived from random effects meta-analysis and reported as weighted mean (95% confidence interval).

RFA, radiofrequency ablation; TFA, transcervical fibroid ablation.

### Temporal trends in fibroid volume, fibroid-related quality of life, and reinterventions

**FIG. 2.**
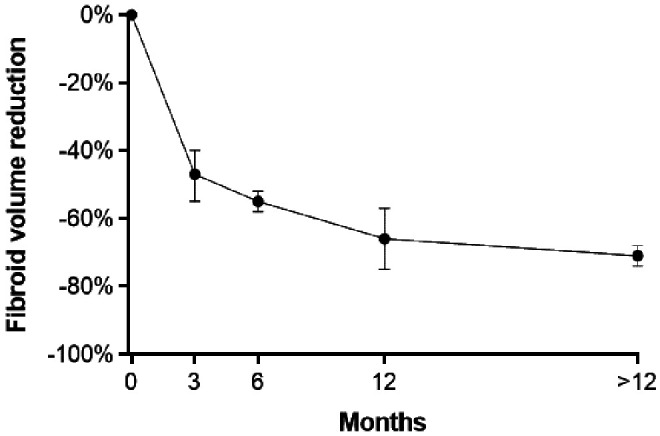
Temporal trends in uterine fibroid volume following radiofrequency ablation of uterine fibroids. Plotted data are mean percent change from baseline and 95% confidence interval. Fibroid volumes at each follow-up interval were significantly smaller than baseline (all *P* < .001). Heterogeneity estimates were *I*^2^ = 54% (*P* = .02) at 3 months, *I*^2^ = 0% (*P* = .44) at 6 months, *I*^2^ = 43% (*P* = .07) at 12 months, and *I*^2^ = 0% (*P* = .42) after 12 months.

**FIG. 3.**
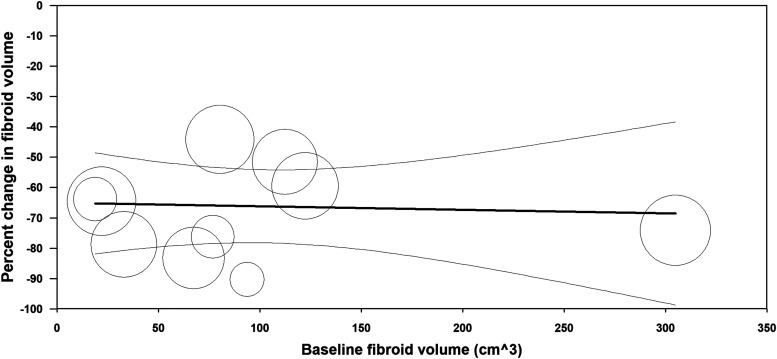
Relationship between uterine fibroid volume at baseline with percent change in uterine volume following radiofrequency ablation. Plotted data are the metaregression line (dark line) and 95% confidence interval (light lines), with results of individual studies denoted by circles, where circle size is proportional to the weighting of the study in the meta-analysis. Percentage of between-study variance explained by baseline fibroid volume (*R*^2^ analog) = 0% (*P* = .83).

Following RFA, mean fibroid volume decreased by 47% at 3 months, 55% at 6 months, 66% at 12 months, and 71% at >12 months follow-up (Fig. 2). Low-to-moderate heterogeneity among studies was observed at each follow-up interval (*I*^2^ values of 54% [*P* = .02], 0% [*P* = .44], 43% [*P* = .07], and 0% [*P* = .42] at 3, 6, 12, and >12 months, respectively). The percent change in fibroid volume 12 months after RFA was consistent across the range of treated fibroid volumes (Fig. 3). In metaregression that adjusted for differences in baseline fibroid volume, using laparoscopic RFA as the reference comparator, fibroid volume reduction was 4% greater with TFA (*P* = .81) and 10% greater with transvaginal RFA (*P* = .47) at 12 months.

**FIG. 4.**
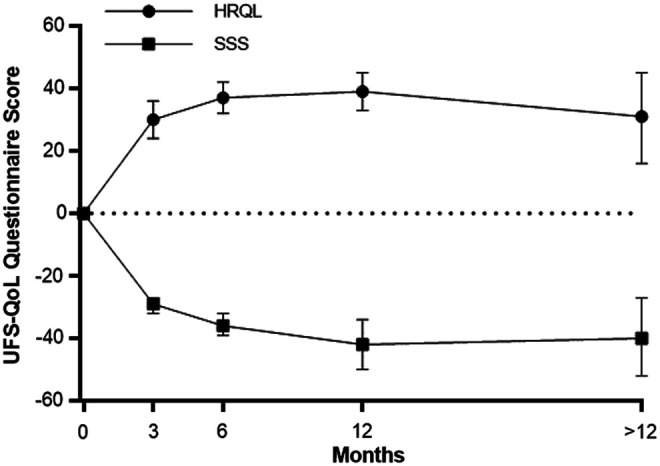
Temporal trends in UFS-QoL subscores following radiofrequency ablation of uterine fibroids. Plotted data are mean absolute change from baseline and 95% confidence interval. HRQL values at each follow-up interval were significantly higher than baseline (all *P* < .001). SSS values at each follow-up interval were significantly lower than baseline (all *P* < .001). Heterogeneity estimates for HRQL were *I*^2^ = 89% (*P* < .001) at 3 months, *I*^2^ = 86% (*P* < .001) at 6 months, *I*^2^ = 91% (*P* < .001) at 12 months, and *I*^2^ = 99% (*P* < .001) after 12 months. Heterogeneity estimates for SSS were *I*^2^ = 46% (*P* = .06) at 3 months, *I*^2^ = 77% (*P* < .001) at 6 months, *I*^2^ = 96% (*P* < .001) at 12 months, and *I*^2^ = 99% (*P* < .001) after 12 months. HRQL, health-related quality of life; SSS, symptom severity score; UFS-QoL, Uterine Fibroid Symptom Health-Related Quality of Life Questionnaire.

**FIG. 5.**
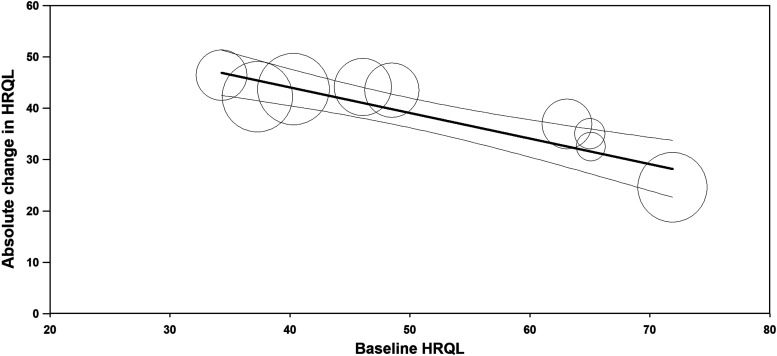
Relationship between HRQL at baseline with HRQL change at 12 months following radiofrequency ablation. Plotted data are the metaregression line (dark line) and 95% confidence interval (light lines), with results of individual studies denoted by circles, where circle size is proportional to the weighting of the study in the meta-analysis. Percentage of between-study variance explained by baseline HRQL (*R*^2^ analog) = 93% (*P* < .001). HRQL, health-related quality of life.

**FIG. 6.**
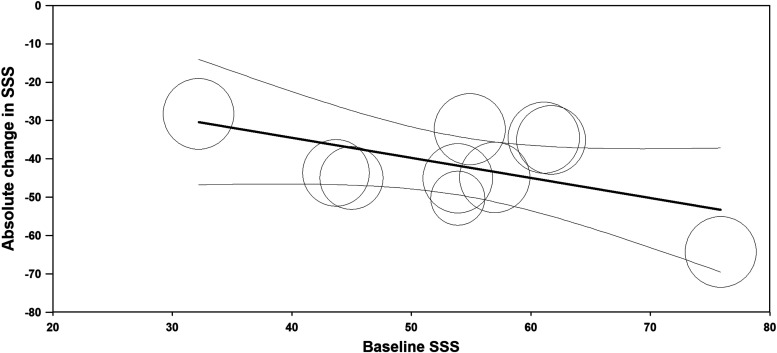
Relationship between SSS at baseline with SSS change at 12 months following radiofrequency ablation. Plotted data are the metaregression line (dark line) and 95% confidence interval (light lines), with results of individual studies denoted by circles, where circle size is proportional to the weighting of the study in the meta-analysis. Percentage of between-study variance explained by baseline SSS (*R*^2^ analog) = 44% (*P* = .05). SSS, symptom severity score.

Quality of life, where higher HRQL scores indicate better quality of life, improved relative to baseline by 30 points at 3 months, 37 points at 6 months, 39 points at 12 months, and 31 points at >12 months follow-up (all *P* < .001 versus baseline). Fibroid symptoms, where lower SSS scores indicate lower symptom severity, decreased by 29, 36, 42, and 40 points relative to baseline over this same period (all *P* < .001 versus baseline) (Fig. 4). Considerable heterogeneity was evident at each follow-up interval for HRQL (*I*^2^ ranged from 86% to 99%, all *P* < .001) and SSS (*I*^2^ ranged from 46% to 99%, all *P* ≤ .06). The heterogeneity in HRQL (Fig. 5) and SSS (Fig. 6) changes was largely explained by the strong inverse association with the baseline value for that variable.

**FIG. 7.**
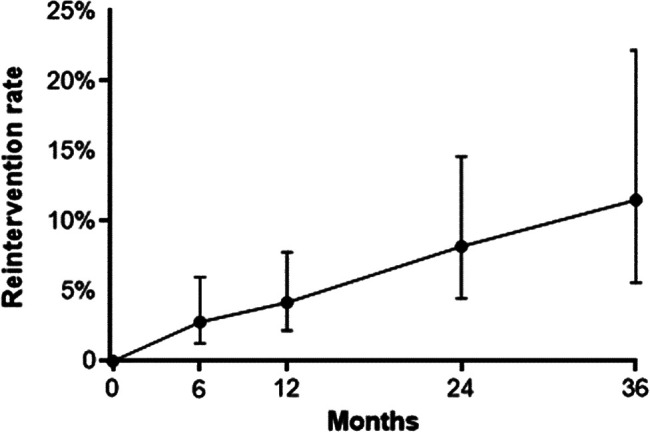
Cumulative reintervention rate following radiofrequency ablation. Error bars are 95% confidence intervals.

The cumulative rate of surgical reinterventions for fibroid-related symptoms was 4.2%, 8.2%, and 11.5% at annual follow-up intervals through 3 years (Fig. 7). The reintervention rate at 12 months was comparable among TFA (2.7%), laparoscopic RFA (3.8%), and transvaginal RFA (5.3%), where *P* ≥ .52 for all pairwise comparisons. The conclusions of this meta-analyses were unchanged among several sensitivity analyses (Supplementary Table S4).

## Discussion

RFA has been used with increasing frequency over the last decade to treat women with uterine fibroids who wish to preserve their uteri and possibly avoid more invasive surgery. Yet there is a paucity of comprehensive reviews regarding RFA of uterine fibroids that synthesize published evidence to help inform women and their gynecologists about typical acute and longer term results. In this systematic review and meta-analysis, the mean RFA procedure time was 49 minutes and performed on an outpatient basis in most cases. Patients returned to normal activities and to work in 5 days, on average, after RFA. We observed significant variability among studies for several outcomes, which was largely attributable to differences in baseline fibroid volume, quality of life, and RFA delivery approaches. Despite this variability, there was strong evidence of sustained fibroid volume reduction, significant improvements in HRQL and SSS, and favorable surgical reintervention rates following RFA.

Several systematic reviews have reported results of RFA for uterine fibroids. Lim et al.^5^ reported that laparoscopic RFA reduced uterine fibroid volume by 81 cm^3^ (*P* < .001), reduced SSS by 43 points (*P* < .001), and improved HRQL by 38 points (*P* < .001), with a reintervention rate of 2.7% over follow-up ranging from 9 to 36 months. More recently, Lin et al.^6^ performed a similar review that included the same RFA studies and ultimately reached the same conclusions. Sandberg et al.^11^ reported a reintervention rate of 0.3% at 1 year and 10.4% at 3 years, reductions in SSS of 37 points at 1 year, and increases in HRQL of 35 points at 1 year after laparoscopic RFA. Taheri et al.^12^ published a review comparing uterine artery embolization, various routes of RFA, and focused ultrasound and found that RFA provided a significantly greater percentage of fibroid volume reduction compared with the other treatments. No previous report has analyzed aggregate outcomes of transvaginal RFA or TFA studies. In this study, we report temporal trends in RFA outcomes, provide comparisons of outcomes by RFA delivery approach, and performed several sensitivity analyses to determine whether the meta-analysis conclusions were robust to various assumptions. Thus, the current review provides novel clinical evidence that arguably represents the most thorough meta-analysis results of RFA for treatment of uterine fibroids.

RFA was used to treat a wide variety of fibroid types and sizes in the included studies. Analysis of fibroid volume decreases in relation to baseline fibroid volumes suggests that RFA provides ∼65% reductions in fibroid volume across a broad range of fibroid sizes. Similarly, despite variation in baseline quality of life scores among studies, RFA provided significant improvements in SSS and HRQL at all follow-up intervals and across the entire range of preprocedural quality-of-life scores.

We also analyzed patient outcomes by RFA delivery approach, which revealed several important observations. First, TFA was associated with a brief mean procedure time, short mean length of stay, and, on average, a faster return to normal activities and work compared with laparoscopic RFA. Transvaginal RFA was also associated with short procedure time and length of stay, but no data related to time to return to normal activities or work were reported. It would be expected that the transvaginal and transcervical routes of RFA would provide similar outcomes, although in theory, TFA may be expected to be the safer of the two options given the need with transvaginal RFA to violate the uterine serosa with a charged electrode or electrode array. Second, RFA delivery approaches were similarly effective in reducing fibroid volume and improving quality of life. Third, surgical reintervention rates for fibroid-related symptoms were favorable after RFA and did not significantly differ among RFA delivery approaches. Furthermore, the rate of reintervention at 3 years was 11.5% in the current review, which favorably compares with reported rates of 17% for uterine artery embolization, 21% for hysteroscopic myomectomy, 24% for endometrial ablation, and 11% for laparoscopic myomectomy over the same period.^13^

Main strengths of this review included adherence to PRISMA guidelines, excellent generalizability of results given the inclusion of almost 1300 patients, and robust conclusions that were unchanged in various sensitivity analyses. There are also several limitations pertaining to the quality of the included studies that may influence conclusions. First, there is less precision in the RFA results after 12 months of follow-up since fewer studies reported longer term data. Second, the number of included studies was insufficient to perform statistical comparisons among RFA delivery approaches for several outcomes. In these cases where we reported the results descriptively, it is plausible that clinically important differences in patient outcomes existed among RFA delivery approaches that were not detectable in our meta-analysis due to insufficient statistical power. Importantly, since the comparative RFA outcomes reported in this study were derived from different studies and analyzed through metaregression, the *post hoc* results should be considered hypothesis generating only. Third, it is not possible to determine from this analysis whether RFA efficacy was influenced by fibroid type or volume due to concerns of aggregation bias whereby real associations observed at the patient level (e.g., fibroid volume in each patient) often do not agree with those observed at the study level (e.g., mean fibroid volume in each study).^14^ Finally, we planned to report the frequency of complications in this meta-analysis. Unfortunately, complication reporting was highly inconsistent and inadequate such that any attempts at reporting these data would have led to inaccurate and misleading results. For example, most articles provided no criteria or definitions regarding complication reporting. Furthermore, several articles simply reported that no complications occurred without any further commentary. Regardless, no serious procedural complications such as death or iatrogenic injury to the bowel, bladder, or ureter were reported in any study. Authors of future RFA studies are encouraged to provide detailed definitions of complications and a complete listing of reported complications during follow-up, with further specification of event seriousness and relationship to the RFA procedure. Lastly, RFA was utilized across a broad range of fibroid types and volumes, suggesting that this therapy is appropriate for most women with symptomatic uterine fibroids.

## Conclusion

RFA of uterine fibroids significantly reduces fibroid volume, provides significant improvements in fibroid-related quality of life, and is associated with favorable reintervention rates.

## Supplementary Material

Supplemental data
