# Supplementary material for: Clinical Performance of Radiofrequency Ablation for Treatment of Uterine Fibroids: Systematic Review and Meta-Analysis of Prospective Studies
Source: J Laparoendosc Adv Surg Tech A. 2019 Nov 8;29(12):1507–17. doi: 10.1089/lap.2019.0550 (PMC7387230; doi:10.1089/lap.2019.0550)
Supplement: Supplemental data [file Supp_Data.pdf]

Supplementary Data

Supplementary Table S1. Medline Search Strategy

Anatomic search terms	
1.	Uterine
2.	Uterus
Diagnosis search terms[a]	
3.	Fibroid
4.	Leiomyoma*
5.	Myoma*
Treatment search terms	
6.	Ablation
7.	Myolysis
8.	Radio frequency
9.	Radiofrequency
10.	Thermal
Combination terms	
11.	Or/1–2
12.	Or/3–5
13.	Or/6–10
14.	And/11–13

[a]An asterisk represents wildcard end-truncation.

Supplementary Table S2. Full-Text Reports Excluded from Meta-Analysis and Primary Reasons for Exclusion

Study	*Main reason for exclusion*
AlHilli et al.[S1]	Endometrial ablation
Bansi-Matharu et al.[S2]	RFA results not reported
Beebeejaun and Varma[S3]	Review article
Bends et al.[S4]	Case report
Berman et al.[S5]	Case report
Berman et al.[S6]	Case series less than 10 patients
Chittawar and Kamath[S7]	Review article
Cho et al.[S8]	Endometrial ablation
Closon and Tulandi[S9]	Review article
Cramer et al.[S10]	Endometrial ablation
Fadare et al.[S11]	Case report
Fadare et al.[S12]	Histologic article
Garza-Leal et al.[S13]	Some patients treated concurrently with hysterectomy; remaining number of patients was less than 10
Gingold et al.[S14]	Review article
Goldfarb[S15]	Review article
Hai and Ding[S16]	Case report
Havryliuk et al.[S17]	Review article
Huirne and Brooks[S18]	Health economics article derived from Brölmann et al.[S19]
Ierardi et al.[S20]	Review article
Ito et al.[S21]	Review article
Jeong et al.[S22]	Case report
Jones et al.[S23]	Review article
Keltz et al.[S24]	Review article
Khan et al.[S25]	Review article
Kim et al.[S26]	Uterine artery embolization performed within 24 hours before RFA
Kubinova et al.[S27]	Review article
Kulkarni et al.[S28]	RFA not performed
Laughlin-Tommaso[S29]	Review article
Lee and Yu[S30]	Review article
Lim et al.[S31]	Review article
Lin et al.[S32]	Review article
Longinotti et al.[S33]	Endometrial ablation
Luo et al.[S34]	Histologic article
Luo et al.[S35]	Histologic article
Milic et al.[S36]	Case series less than 10 patients
Munro[S37]	Case report
Ouldamer and Marret[S38]	Review article
Quinn and Gedroyc[S39]	Review article
Recaldini et al.[S40]	Case series less than 10 patients
Rubino et al.[S41]	Endometrial ablation
Sandberg et al.[S42]	Review article
Shen et al.[S43]	Review article
Stein and Ascher-Walsh[S44]	Review article
Shu et al.[S45]	Histologic article
Taheri et al.[S46]	Review article
Thompson and Carr[S47]	Review article
Toub[S48]	Review article
Van der Kooij et al.[S49]	Review article
Varon et al.[S50]	Case report
Walter et al.[S51]	Histologic article derived from Brucker, 2014[S52]
Yin et al.[S53]	Retrospective enrollment

RFA, radiofrequency ablation.

Supplementary Table S3. Study Quality Assessment

Study	*1*	*2*	*3*	*4*	*5*	*6*	*7*	*8*	*9*	*10*	*11*	*12*	*Quality rating*
Bongers et al.[S54]	Y	Y	Y	Y	Y	Y	Y	N	N	Y	N	NA	Good
Braun et al.[S55]	Y	Y	Y	Y	N	Y	N	N	N	N	N	NA	Poor
Brucker et al.[S52]	Y	Y	Y	Y	Y	Y	Y	N	N	Y	N	NA	Good
Carrafiello et al.[S56]	Y	Y	Y	Y	N	Y	Y	N	N	N	N	NA	Fair
Cho et al.[S57]	Y	Y	Y	Y	N	Y	Y	N	N	N	N	NA	Fair
Cho et al.[S58]	Y	Y	Y	Y	N	Y	Y	N	N	N	N	NA	Fair
Chudnoff et al.[S59]	Y	Y	Y	Y	Y	Y	Y	N	N	Y	N	NA	Good
Chudnoff et al.[S60]	Y	Y	Y	Y	Y	Y	Y	N	N	Y	N	NA	Good
Galen et al.[S61]	Y	Y	Y	Y	N	Y	Y	N	N	Y	N	NA	Fair
Ghezzi et al.[S62]	Y	Y	Y	Y	N	Y	Y	N	N	N	N	NA	Fair
Iversen and Dueholm[S63]	Y	Y	Y	Y	N	Y	Y	N	N	Y	N	NA	Fair
Jiang et al.[S64]	Y	Y	Y	Y	N	Y	Y	N	N	N	N	NA	Fair
Kim et al.[S65]	Y	Y	Y	Y	N	Y	Y	N	N	Y	N	NA	Fair
Lee et al.[S66]	Y	Y	Y	Y	N	Y	Y	N	N	N	N	NA	Fair
Marcos et al.[S67]	Y	Y	Y	Y	N	Y	Y	N	N	Y	N	NA	Fair
Meng et al.[S68]	Y	Y	Y	Y	N	Y	N	N	N	Y	N	NA	Fair
Rattray et al.[S69]	Y	Y	Y	Y	Y	Y	Y	N	N	Y	N	NA	Good
Rey et al.[S70]	Y	Y	Y	Y	N	Y	Y	N	N	Y	N	NA	Fair
Turtuliciet al.[S71]	Y	Y	Y	Y	N	Y	Y	N	Y	N	N	NA	Fair
Wu et al.[S72]	Y	Y	Y	Y	N	Y	Y	N	N	N	N	NA	Fair

Methodological quality of studies assessed with the National Institute of Health assessment tool for before/after studies. Item numbers and associated descriptions include: (1) objective clearly stated; (2) eligibility criteria described; (3) representative patient population; (4) all eligible participants enrolled in study; (5) sufficient sample size; (6) intervention described; (7) outcome measures specified; (8) outcome assessors blinded; (9) loss to follow-up and intention-to-treat analysis; (10) statistical analysis of outcome measures before and after intervention; (11) interrupted time-series design; (12) individual data used for group-level effects.

N, no; NA, not applicable; Y, yes.

Supplement Table S4. Sensitivity Analyses

Outcome	*Primary analysis (random-effects model)*	*Sensitivity analysis (fixed-effects model)*	*Sensitivity analysis (one-study removed analysis)*		*Sensitivity analysis (high-quality studies)*[a]
			Minimum	*Maximum*	
Procedure time, minutes[b]	49 (41 to 56)	34 (34 to 34)	43 (36 to 51)	51 (42 to 59)	70 (47 to 92)
Time to discharge, hours[b]	8.2 (6.3 to 10)	2.8 (2.7 to 2.9)	6.1 (4.9 to 7.2)	9.3 (4.1 to 14.6)	6.3 (2.1 to 10.5)
Time to return to normal activities (days)[b]	5.2 (3.3 to 7.1)	3.1 (2.8 to 3.4)	4.2 (2.4 to 6.1)	6.1 (4.2 to 8.0)	5.9 (3.2 to 8.7)
Time to return to work (days)[b]	5.1 (3.7 to 6.5)	3.9 (3.6 to 4.3)	4.0 (3.4 to 4.6)	6.5 (3.8 to 9.2)	6.4 (3.6 to 9.3)
Fibroid volume percent change[c]	−66% (−75% to −57%)[d]	−65% (−72% to −59%)[d]	−69% (−76% to −62%)[d]	−64% (−73% to −55%)[d]	−56% (−71% to −41%)[d]
SSS change[c]	−42 (−50 to −34)[d]	−40 (−42 to −39)[d]	−44 (−52 to −35)[d]	−39 (−43 to −34)[d]	−34 (−36 to −31)[d]
HRQL change[c]	39 (33 to 45)[d]	39 (37 to 41)[d]	38 (32 to 44)[d]	42 (40 to 45)[d]	43 (41 to 46)[d]
Reintervention rate[e]	4.2% (2.2% to 7.8%)	5.5% (3.9% to 7.7%)	3.6% (2.2% to 6.0%)	4.9% (2.7% to 8.9%)	2.3% (0.6% to 8.7%)

[a]Analysis includes only studies with a study quality of good using the National Institute of Health assessment tool for before/after studies.
[b]Values are weighted mean (95% confidence interval).
[c]Values are weighted mean difference of change from baseline to 12 months (95% confidence interval).
[d]$p < 0.001$ relative to baseline.
[e]Values are weighted reintervention rate through 12 months (95% confidence interval).
HRQL, health-related quality of life; SSS, symptom severity score.

Supplementary References

S1. Alhilli MM, Wall DJ, Brown DL, Weaver AL, Hopkins MR, Famuyide AO. Uterine ultrasound findings after radiofrequency endometrial ablation: Correlation with symptoms. Ultrasound Q 2012;28:261–268.

S2. Bansi-Matharu L, Gurol-Urganci I, Mahmood TA, Templeton A, Van Der Meulen JH, Cromwell DA. Rates of subsequent surgery following endometrial ablation among English women with menorrhagia: Population-based cohort study. BJOG 2013;120:1500–1507.

S3. Beebeejaun Y, Varma R. Heavy menstrual flow: Current and future trends in management. Rev Obstet Gynecol 2013;6:155–164.

S4. Bends R, Toub DB, Romer T. Normal spontaneous vaginal delivery after transcervical radiofrequency ablation of uterine fibroids: A case report. Int J Womens Health 2018;10:367–369.

S5. Berman JM, Puscheck EE, Diamond MP. Full-term vaginal live birth after laparoscopic radiofrequency ablation of a large, symptomatic intramural fibroid: A case report. J Reprod Med 2012;57:159–163.

S6. Berman JM, Bolnick JM, Pemueller RR, Garza Leal JG. Reproductive outcomes in women following radiofrequency volumetric thermal ablation of symptomatic fibroids. A retrospective case series analysis. J Reprod Med 2015;60:194–198.

S7. Chittawar PB, Kamath MS. Review of nonsurgical/minimally invasive treatments and open myomectomy for uterine fibroids. Curr Opin Obstet Gynecol 2015;27:391–397.

S8. Cho EA, Um MJ, Kim SA, Kim SJ, Jung H. Comparison of laparoscopic radiofrequency myolysis (LRFM) and ultrasonographic radiofrequency myolysis (URFM) in treatment of midline dysmenorrhea. J Menopausal Med 2014;20:75–79.

S9. Closon F, Tulandi T. Uterine myomata: Organ-preserving surgery. Best Pract Res Clin Obstet Gynaecol 2016; 35:30–36.

S10. Cramer MS, Klebanoff JS, Hoffman MK. Pain is an independent risk factor for failed global endometrial ablation. J Minim Invasive Gynecol 2018;25:1018–1023.

S11. Fadare O, Qin L, Martel M, Tavassoli FA. Pathology of the NovaSure (radio-frequency) impedance-controlled endometrial ablation system. Arch Pathol Lab Med 2005;129:1175–1178.

S12. Fadare O, Wang SA, Renshaw IL. Does the radiofrequency impedance-controlled endometrial ablation have any morphologic effects on uterine leiomyomata? Report of 3 cases. Diagn Pathol 2008;3:28.

S13. Garza-Leal JG, Toub D, León IH, et al. Transcervical, intrauterine ultrasound-guided radiofrequency ablation of uterine fibroids with the VizAblate System: Safety, tolerability, and ablation results in a closed abdomen setting. Gynecol Surg 2011;8:327–334.

S14. Gingold JA, Gueye NA, Falcone T. Minimally invasive approaches to myoma management. J Minim Invasive Gynecol 2018;25:237–250.

S15. Goldfarb HA. Myolysis revisited. JSLS 2008;12:426–430.

S16. Hai N, Ding X. Intrauterine adhesion after transvaginal ultrasound-guided radiofrequency myolysis. J Obstet Gynaecol Res 2015;41:1851–1854.

S17. Havryliuk Y, Setton R, Carlow JJ, Shaktman BD. Symptomatic fibroid management: Systematic review of the literature. JSLS 2017;21.

S18. Huirne J, Brooks E. Improvement in health utility after transcervical radiofrequency ablation of uterine fibroids with the sonata system: Health utility after radiofrequency ablation. Eur J Obstet Gynecol Reprod Biol 2018; 224:175–180.

S19. Brölmann H, Bongers M, Gaarza-Leal JG, et al. The FAST-EU trial: 12-month clinical outcomes of women after intrauterine sonography-guided transcervical radiofrequency ablation of uterine fibroids. Gynecol Surg 2016;13:27–35.

S20. Ierardi AM, Savasi V, Angileri SA, et al. Percutaneous high frequency microwave ablation of uterine fibroids: Systematic review. Biomed Res Int 2018;2018:2360107.

S21. Ito T, Mattingly PJ, Jan AG, Biscette SM, Kim JHJ. Alternatives to excisional therapy: A clinical review of our current options to conservatively manage symptomatic leiomyomas. Curr Opin Obstet Gynecol 2018;30:279–286.

S22. Jeong HJ, Kwon BS, Choi YJ, Huh CY. Rectouterine fistula after laparoscopic ultrasound-guided radiofrequency ablation of a uterine fibroid. Obstet Gynecol Sci 2014;57:553–556.

S23. Jones S, O'donovan P, Toub D. Radiofrequency ablation for treatment of symptomatic uterine fibroids. Obstet Gynecol Int 2012;2012:194839.

S24. Keltz J, Levie M, Chudnoff S. Pregnancy outcomes after direct uterine myoma thermal ablation: Review of the literature. J Minim Invasive Gynecol 2017;24:538–545.

S25. Khan AT, Shehmar M, Gupta JK. Uterine fibroids: Current perspectives. Int J Womens Health 2014;6:95–114.

S26. Kim HS, Tsai J, Jacobs MA, Kamel IR. Percutaneous image-guided radiofrequency thermal ablation for large symptomatic uterine leiomyomata after uterine artery embolization: A feasibility and safety study. J Vasc Interv Radiol 2007;18:41–48.

S27. Kubinova K, Mara M, Horak P, Kriz R, Maskova J, Kuzel D. [News and perspectives in uterine fibroids radiotherapy]. Ceska Gynekol 2009;74:22–26.

S28. Kulkarni MR, Dutta I, Dutta DK. Clinicopathological study of uterine leiomyomas: A multicentric study in rural population. J Obstet Gynaecol India 2016;66:412–416.

S29. Laughlin-Tommaso SK. Non-surgical management of myomas. J Minim Invasive Gynecol 2018;25:229–236.

S30. Lee BB, Yu SP. Radiofrequency ablation of uterine fibroids: A review. Curr Obstet Gynecol Rep 2016;5:318–324.

S31. Lim KJ, Yoon DY, Kim JH, et al. Percutaneous radiofrequency ablation for symptomatic uterine leiomyomas: A systematic review and meta-analysis. Clin Exp Obstet Gynecol 2016;43:643–649.

S32. Lin L, Ma H, Wang J, et al. Quality of life, adverse events, and reintervention outcomes after laparoscopic radiofrequency ablation for symptomatic uterine fibroids: A meta-analysis. J Minim Invasive Gynecol 2019;26:409–416.

S33. Longinotti MK, Jacobson GF, Hung YY, Learman LA. Probability of hysterectomy after endometrial ablation. Obstet Gynecol 2008;112:1214–1220.

S34. Luo X, Shen Y, Song WX, Chen PW, Xie XM, Wang XY. Pathologic evaluation of uterine leiomyoma treated with radiofrequency ablation. Int J Gynaecol Obstet 2007; 99:9–13.

S35. Luo X, Shu SR, Ma XF, Shuai HL. The research of feasibility and efficacy of radiofrequency ablation in treating uterine fibroids. Medicine (Baltimore) 2015;94:e1956.

S36. Milic A, Asch MR, Hawrylyshyn PA, et al. Laparoscopic ultrasound-guided radiofrequency ablation of uterine fibroids. Cardiovasc Intervent Radiol 2006;29:694–698.
S37. Munro MG. Hysteroscopic myomectomy of FIGO type 2 leiomyomas under local anesthesia: Bipolar radiofrequency needle-based release followed by electromechanical morcellation. J Minim Invasive Gynecol 2016;23:12–13.
S38. Ouldamer L, Marret H. [Therapeutic alternatives of uterine fibroids except medicinal treatment and embolization]. J Gynecol Obstet Biol Reprod (Paris) 2011;40:928–936.
S39. Quinn SD, Gedroyc WM. Thermal ablative treatment of uterine fibroids. Int J Hyperthermia 2015;31:272–279.
S40. Recaldini C, Carrafiello G, Lagana D, et al. Percutaneous sonographically guided radiofrequency ablation of medium-sized fibroids: Feasibility study. AJR Am J Roentgenol 2007;189:1303–1306.
S41. Rubino RJ, Roy KH, Presthus J, Trupin S. Abnormal uterine bleeding control by sequential application of hysteroscopic lesion morcellation and endometrial ablation. J Reprod Med 2017;62:102–110.
S42. Sandberg EM, Tummers F, Cohen SL, Van Den Haak L, Dekkers OM, Jansen FW. Reintervention risk and quality of life outcomes after uterine-sparing interventions for fibroids: A systematic review and meta-analysis. Fertil Steril 2018;109:698–707 e1.
S43. Shen SH, Fennessy F, Mcdannold N, Jolesz F, Tempany C. Image-guided thermal therapy of uterine fibroids. Semin Ultrasound CT MR 2009;30:91–104.
S44. Stein K, Ascher-Walsh C. A comprehensive approach to the treatment of uterine leiomyomata. Mt Sinai J Med 2009;76:546–556.
S45. Shu SR, Luo X, Song WX, Chen PW. Ultra-structure changes and survivin expression in uterine fibroids after radiofrequency ablation. Int J Hyperthermia 2015;31:896–899.
S46. Taheri M, Galo L, Potts C, Sakhel K, Quinn SD. Nonresective treatments for uterine fibroids: A systematic review of uterine and fibroid volume reductions. Int J Hyperthermia 2019;36:295–301.
S47. Thompson MJ, Carr BR. Intramural myomas: To treat or not to treat. Int J Womens Health 2016;8:145–149.
S48. Toub DB. A new paradigm for uterine fibroid treatment: Transcervical, intrauterine sonography-guided radiofrequency ablation of uterine fibroids with the sonata system. Curr Obstet Gynecol Rep 2017;6:67–73.
S49. Van Der Kooij SM, Ankum WM, Hehenkamp WJ. Review of nonsurgical/minimally invasive treatments for uterine fibroids. Curr Opin Obstet Gynecol 2012;24:368–375.
S50. Varon S, Parvataneni R, Waetjen E, Dunn K, Jacoby VL. Misdiagnosis of leiomyosarcoma after radiofrequency ablation of uterine myomas. J Minim Invasive Gynecol 2019;26:564–566.
S51. Walter CB, Hartkopf AD, Schoeller D, et al. Ultrasound guided core needle biopsy prior to thermo ablative treatment of uterine tumors: First results. Arch Gynecol Obstet 2018;297:387–392.
S52. Brucker SY, Hahn M, Kraemer D, Taran FA, Isaacson KB, Kramer B. Laparoscopic radiofrequency volumetric thermal ablation of fibroids versus laparoscopic myomectomy. Int J Gynaecol Obstet 2014;125:261–265.
S53. Yin G, Chen M, Yang S, Li J, Zhu T, Zhao X. Treatment of uterine myomas by radiofrequency thermal ablation: A 10-year retrospective cohort study. Reprod Sci 2015; 22:609–614.
S54. Bongers M, Brolmann H, Gupta J, Garza-Leal JG, Toub D. Transcervical, intrauterine ultrasound-guided radiofrequency ablation of uterine fibroids with the VizAblate(R) System: Three- and six-month endpoint results from the FAST-EU study. Gynecol Surg 2015; 12:61–70.
S55. Braun KM, Sheridan M, Latif EZ, et al. Surgeons' early experience with the Acessa procedure: Gaining proficiency with new technology. Int J Womens Health 2016;8:669–675.
S56. Carrafiello G, Recaldini C, Fontana F, et al. Ultrasound-guided radiofrequency thermal ablation of uterine fibroids: Medium-term follow-up. Cardiovasc Intervent Radiol 2010;33:113–119.
S57. Cho HH, Kim JH, Kim MR. Transvaginal radiofrequency thermal ablation: A day-care approach to symptomatic uterine myomas. Aust N Z J Obstet Gynaecol 2008;48:296–301.
S58. Cho HH, Kim MR, Kim JH. Outpatient multimodality management of large submucosal myomas using transvaginal radiofrequency myolysis. J Minim Invasive Gynecol 2014;21:1049–1054.
S59. Chudnoff SG, Berman JM, Levine DJ, Harris M, Guido RS, Banks E. Outpatient procedure for the treatment and relief of symptomatic uterine myomas. Obstet Gynecol 2013;121:1075–1082.
S60. Chudnoff S, Guido R, Roy K, Levine D, Mihalov L, Garza Leal JG. Ultrasound-guided transcervical ablation of uterine leiomyomata. Obstet Gynecol 2019;133: 13–22.
S61. Galen DI, Pemueller RR, Leal JG, Abbott KR, Falls JL, Macer J. Laparoscopic radiofrequency fibroid ablation: Phase II and phase III results. JSLS 2014;18:182–190.
S62. Ghezzi F, Cromi A, Bergamini V, Scarperi S, Bolis P, Franchi M. Midterm outcome of radiofrequency thermal ablation for symptomatic uterine myomas. Surg Endosc 2007;21:2081–2085.
S63. Iversen H, Dueholm M. Radiofrequency thermal ablation for uterine myomas: Long-term clinical outcomes and reinterventions. J Minim Invasive Gynecol 2017;24:1020–1028.
S64. Jiang X, Thapa A, Lu J, Bhujohory VS, Liu Y, Qiao S. Ultrasound-guided transvaginal radiofrequency myolysis for symptomatic uterine myomas. Eur J Obstet Gynecol Reprod Biol 2014;177:38–43.
S65. Kim CH, Kim SR, Lee HA, Kim SH, Chae HD, Kang BM. Transvaginal ultrasound-guided radiofrequency myolysis for uterine myomas. Hum Reprod 2011;26:559–563.
S66. Lee Y, Cho HH, Kim JH, et al. Radiofrequency thermal ablation of submucosal leiomyoma: A preliminary report on health, symptom, and quality of life outcomes. J Gynecol Surg 2010;26:227–231.
S67. Marcos RG, Monleón J, Martínez-Varea A, et al. Percutaneous ultrasound-guided radiofrequency thermal ablaiton for treatment of uterine fibroids. Open J Obstet Gynecol 2014;4:716–724.
S68. Meng X, He G, Zhang J, et al. A comparative study of fibroid ablation rates using radio frequency or high-intensity focused ultrasound. Cardiovasc Intervent Radiol 2010;33:794–799.
S69. Rattray DD, Weins L, Regush LC, Bowen JM, O'reilly D, Thiel JA. Clinical outcomes and health care utilization

pre- and post-laparoscopic radiofrequency ablation of symptomatic fibroids and laparoscopic myomectomy: A randomized trial of uterine-sparing techniques (TRUST) in Canada. Clinicoecon Outcomes Res 2018;10:201–212.
S70. Rey VE, Labrador R, Falcon M, Garcia-Benitez JL. Transvaginal radiofrequency ablation of myomas: Technique, outcomes, and complications. J Laparoendosc Adv Surg Tech A 2019;29:24–28.
S71. Turtulici G, Orlandi D, Dedone G, et al. Ultrasound-guided transvaginal radiofrequency ablation of uterine fibroids assisted by virtual needle tracking system: A preliminary study. Int J Hyperthermia 2019;35:97–104.
S72. Wu XJ, Guo Q, Cao BS, et al. Uterine leiomyomas: Safety and efficacy of US-guided suprapubic transvaginal radiofrequency ablation at 1-year follow-up. Radiology 2016;279:952–960.
